# Freeze–Thaw Damage Characterization of Cement-Stabilized Crushed Stone Base with Skeleton Dense Gradation

**DOI:** 10.3390/ma17061228

**Published:** 2024-03-07

**Authors:** Rui Xiao, Baoping An, Fei Wu, Wensheng Wang, Yi Sui, Yinghan Wang

**Affiliations:** 1 Gansu Province Transportation Planning Survey & Design Institute Co., Ltd., Lanzhou 730030, China; xiaorui_gansu@outlook.com; 2 College of Transportation, Jilin University, Changchun 130025, China; wufei1719@mails.jlu.edu.cn (F.W.); suiyi1721@mails.jlu.edu.cn (Y.S.); wangyh_jlu@outlook.com (Y.W.)

**Keywords:** cement-stabilized crushed stone, freeze–thaw damage, mechanical performance, ultrasonic velocity, acoustic emission

## Abstract

The skeleton dense graded cement-stabilized crushed stone base is a widely used material for road construction. However, this material is susceptible to freeze–thaw damage, which can lead to degradation and failure, for which there is still a lack of an in-depth understanding of the freeze–thaw damage characteristics. This study aims to assess the mechanical performance and the freeze–thaw damage characteristics of the cement-stabilized crushed stone base with skeleton dense gradation based on a mechanical test and acoustic technology in a laboratory. There is a gradually increasing trend in the mass loss rate of the base material with an increase in freeze–thaw cycles. The curve steepens significantly after 15 cycles, following a parabola-fitting pattern relationship. The compressive strength of the cement-stabilized crushed stone base also decreased with a parabola-fitting pattern, and the decrease rate may accelerate as the freeze–thaw cycles increase. The resilience modulus of the base material decreased with increasing freeze–thaw cycles, following a parabolic trend. This suggests that the material’s resistance to freeze–thaw damage decreases with increasing cycles. The ultrasonic wave velocity decreased with increasing freeze–thaw cycles, exhibiting a parabolic trend. This decline can be attributed to microcracks and defects developing within the material, offering insights for monitoring and predicting its service life. The damage progression of the cement-stabilized crushed stone base was found to occur in three stages: initial, stationary, and failure. The duration of stage I increased with freeze–thaw cycles, while the duration of stage III decreased. The findings provide valuable insights into the mechanisms and processes of freeze–thaw damage in a cement-stabilized crushed stone base with skeleton dense gradation.

## 1. Introduction

In the field of civil engineering, the durability and stability of road bases are essential factors for ensuring the longevity and safety of road infrastructure [1,2]. A crushed stone base is a widely used material for road construction due to its durability and cost-effectiveness, which is commonly used as a subbase material [3,4]. However, this material is susceptible to freeze–thaw damage, which can lead to a decline in the mechanical properties and durability of the road base, ultimately leading to premature failure [5,6,7]. Freeze–thaw damage occurs when water trapped within the material freezes during cold weather, expands, and thaws during warmer periods. This process results in microstructural changes that can compromise the material’s integrity [8]. To counteract this, cement and gradation designs are often used to stabilize the crushed stone base, enhancing its resistance to the freezing–thawing effect [9]. Nevertheless, the effect of the skeleton dense gradation for cement-stabilized base material on its freeze–thaw damage characteristics remains elusive.

Freeze–thaw damage has a significant impact on the strength and durability of a cement-stabilized macadam base [10,11]. Understanding the mechanisms of freeze–thaw damage in the cement-stabilized macadam base materials is the premise for identifying potential strategies to enhance their durability. To achieve this, a combination of experimental and numerical methods will be employed, encompassing both laboratory testing and theoretical modeling. Previous studies have shown that the road performance and other properties of the cement-stabilized macadam base decrease with each additional freezing–thawing cycle [12]. A series of experiments have been conducted to simulate freezing–thawing cycles and analyze the physical properties of a cement-stabilized crushed stone base, which focused on measuring changes in compressive strength, the modulus of elasticity, permeability, and so on under freeze–thaw conditions. The study reveals that cement content affects the temperature, water content, and deformation of cement-stabilized soil during freeze–thaw cycles [13]. Adding cement reduces deformation mainly due to water reduction and structure compaction. Wang et al. found that adding cement significantly improves its compaction performance, but under intense freezing and thawing, its strength decreases, and they recommended 5% cement content for high-speed railway subgrade in cold regions [14]. Li et al. showed that both cement and micro-silica can improve the strength and durability of saline soil. Micro-silica, in particular, provides better freeze–thaw cycle durability [15]. Mixtures of cement and micro-silica achieve superior performance compared to cement or micro-silica alone. Rhardane et al. adopted the numerical methodology at the microscale to quantify the freezing–thawing damage mechanisms on cementitious materials [16]. They found that the most harmful factors are supercooling and delayed ice nucleation, and the water-to-cement ratio and minimum temperature during freezing–thawing cycles also contribute to material deterioration, but air entrainment can reduce the damage. Mardani-Aghabaglou et al. found that the combined effect of sulfate attack and freezing–thawing action on cement-stabilized clay is significant, leading to a decrease in strength and an increase in permeability. Using sulfate-resistant cement is more effective than ordinary Portland cement in resisting these effects [17].

In order to improve the freezing resistance of base materials, some studies mainly focused on selecting modified materials including fibers, solid wastes, etc., and preparation parameters [18,19,20]. Wang et al. prepared cement-stabilized gravel with 5 wt% cement and emulsified asphalt, which performed well in both mechanics and frost resistance [21]. Emulsified asphalt can improve permeability, extend the strength loss process, and mitigate frost heave stress. It can also slow down the generation speed of micro cracks. Ding et al. pointed out that the addition of fiber can improve the unconfined compressive strength of fiber and cement-stabilized soil and proposed an empirical model for predicting unconfined compressive strength [22]. More and more solid wastes were introduced to road infrastructure construction. Ding et al. revealed that freeze–thaw cycles degrade the mechanical properties of tailings reinforced with cement-based materials. However, the frozen temperature does not significantly affect the unconfined compressive strength [23]. Ying et al. pointed out that marine shell powder can improve the mechanical properties of cement-solidified coastal clay to some extent, and the corresponding best marine shell powder content is 15%, which can enhance the compactness of coastal cement clay under freeze–thaw cycles [24]. A mathematical empirical model was also established to predict the strength of coastal cement clay modified by seashell powder. Zhang et al. designed the low-frost-susceptible base material and used chemical stabilization with fly ash, cement, and fibers to improve soil frost resistance [25]. However, the low-frost-susceptible base material may have a negative impact on the compaction effect of the pavement base, increase the project cost, and lead to the intensification of structural stiffness differentiation.

Meanwhile, the application of non-destructive testing (NDT) techniques is essential for the evaluation of freeze–thaw damage in the cement-stabilized macadam base materials. NDT methods like ultrasonic testing and acoustic emissions provide valuable insights into the internal structure and potential damage without causing harm to the material [26,27]. El-Mir, A. found that the residual compressive strength of self-consolidating concrete can be accurately estimated using UPV measurements before and after being subjected to 400 F–T cycles [28]. Liu et al. studied cement-stabilized coral aggregate, which has potential as a sustainable base material for roads and airports on islands. Its bending fracture characteristics were studied using acoustic emission technology, revealing brittleness and tensile cracking dominance in the material [29]. Conventional destructive testing methods can provide valuable insights but are limited by their destructive nature. These NDT techniques possess effectiveness in characterizing freeze–thaw damage in cement-stabilized crushed stone bases with skeleton dense gradation.

The objective of this study is to investigate the freeze–thaw damage characteristics of the cement-stabilized crushed stone base with skeleton dense gradation. To achieve these objectives, a series of experiments will be conducted to simulate freezing–thawing cycles and analyze the mechanical properties of the cement-stabilized crushed stone base. The experiments will focus on measuring changes in the compressive strength and resilience modulus under freeze–thaw conditions. In addition, non-destructive testing methods such as ultrasonic testing and acoustic emission technology will be employed to assess the internal damage and identify potential damage processes. Previous studies may have focused on general materials or specific aspects of freeze–thaw damage. The innovativeness of this study lies in the comprehensive analysis of the freeze–thaw damage process, considering the unique properties of cement-stabilized crushed stone with skeleton dense gradation. The findings from this study not only contribute to our understanding of damage mechanisms but also contribute to more precise life predictions of road structures by damage characteristics, leading to cost-effective maintenance strategies and longer-lasting infrastructure.

## 2. Materials and Methods

### 2.1. Experimental Materials

As a cementing material, cement is the main reason for the strength formation of cement-stabilized macadam base material. In this study, P.O 42.5 ordinary Portland cement was selected as the cementing material. Its performance indexes are shown in Table 1, which meets the technical requirements and are similar to the existing literature [21], indicating the accuracy and rationality of the test results.

Aggregate is an important component of cement-stabilized macadam base material, which can be divided into coarse aggregate and fine aggregate according to its particle size. Usually, a particle size of 4.75 mm is used as the boundary size between coarse aggregates and fine aggregates. Coarse aggregate is the skeleton of cement-stabilized macadam base material, and fine aggregate and cement are mixed to fill the skeleton to form a whole. Moreover, fine aggregate exhibits similar characteristics, including liquid and plastic limits, and a plasticity index referring to the previous literature [21]. In this study, considering the strength of the parent rock, basalt rock was selected as aggregate, and the performance properties of coarse aggregate and fine aggregate are shown in Table 2 and Table 3. The particle size distribution of each grade of aggregate and its screening results are shown in Table 4.

### 2.2. Material Gradations and Specimen Preparation

**Figure 1:**
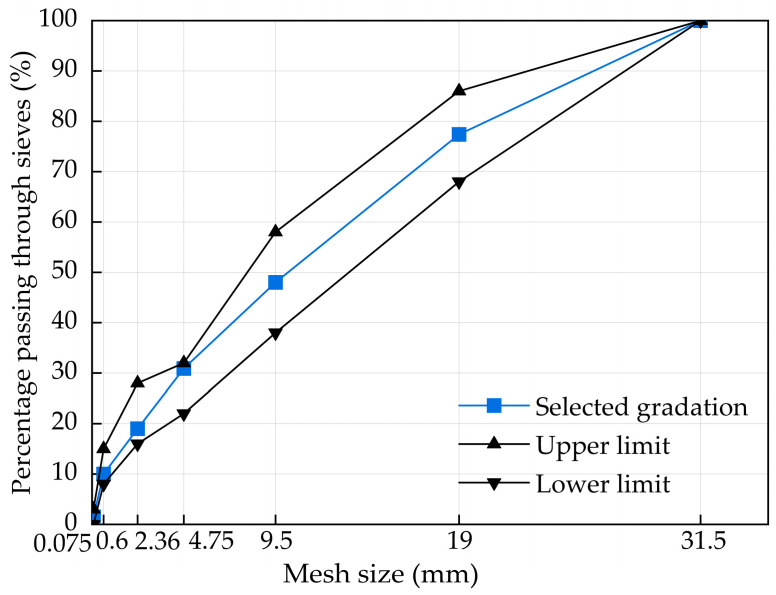
The cement-stabilized macadam base gradation in this study.

In this study, the cement-stabilized macadam base material with skeleton dense gradation was designed. The gradation curves of skeleton dense gradation are shown in Figure 1. The cement content is 5% and the compaction degree is 98%. Specimens are prepared by static pressure molding, and the maximum dry density and optimum water content of skeleton dense gradation are obtained. For the cement-stabilized macadam base materials with skeleton dense gradation, the maximum dry density is 2.384 g/cm^3^ and the optimum moisture content is 4.7%. According to the Test Methods of Materials Stabilized with Inorganic Binders for Highway Engineering (JTG E 51-2009) [31], the diameter–height ratio of the specified specimen is generally 1:1, and the recommended test size is Φ150 mm × 150 mm, Φ100 mm × 100 mm, or Φ50 mm × 50 mm according to the aggregate size. In this study, a Φ150 mm × 150 mm cylindrical specimen was prepared using static pressure molding. Based on the maximum dry density and optimum moisture content of skeleton dense gradation, the quality of the experimental specimens is calculated and configured. The wrapped specimens were put into the standard curing room, in which the curing temperature was (20 ± 2) °C and the relative humidity was above 95%. The specimen was cured for 28 days and placed in water on the last day of curing, of which the water surface was 2.5 cm high from the top surface. Then, a series of tests such as freeze–thaw and unconfined compressive strength was carried out after curing.

### 2.3. Experimental Methods

#### 2.3.1. Freeze–Thaw Cycle Test

In this study, the freeze–thaw cycle test adopted the high–low temperature-alternating damp-heat test box to control the temperature. According to the frost-resistance test requirements in “Test Methods of Materials Stabilized with Inorganic Binders for Highway Engineering” (JTG E 51-2009) [31], the specimen was placed in water after 27 days of curing, and then it was taken out of the water on the 28th day and the surface moisture was dried. The freeze–thaw cycle test was carried out after the specimen was numbered, and the freeze–thaw temperatures were set at −18 °C (freezing time of 16 h) and 20 °C (melting time of 8 h), with at least a 20 mm gap between specimens to ensure the freeze–thaw effect. As for the determination of freeze–thaw cycles, according to the research results of the previous study [32], the characteristics of the northeast seasonal frozen area are that the average temperature in the coldest months is approximately −8 °C~−20 °C, the annual average freeze–thaw cycles are less than 150, and the indoor equivalent freeze–thaw cycles are approximately 10. Referring to the previous studies [21,33], the number of freeze–thaw cycles was selected as 0~20. In order to analyze the damage changes in freeze–thaw cycles to cement-stabilized macadam base materials, this study chose 0, 5, 10, 15, and 20 freeze–thaw cycles. This process is repeated for a certain number of times according to the requirements of the test specification. After a certain number of freezing and thawing cycles, the specimen is removed from the freezing and thawing device, measuring its mass and recording the data. Meanwhile, it is observed whether there is any significant change in appearance or cracking in the specimens after a certain number of freezing and thawing cycles.

#### 2.3.2. Mechanical Performance Test

The unconfined compressive mechanical test is carried out with reference to the requirements of Chinese specification (JTG E 51-2009) [31]. The initial weight, length, and height of each experimental specimen were recorded. We then placed the specimens in an electro hydraulic servo universal testing machine that meets the requirements of the test specification. Then, we started the compressive strength testing machine and compressed the specimens at a loading rate of 1 mm/min until failure. Finally, the compressive strength data were recorded and analyzed to calculate the compressive strength (*R*) of cement-stabilized crushed stone base (*R* = *P*/*A*), evaluating its compressive strength performance. *P* is the maximum force when the specimen is damaged (N) and *A* is the cross-sectional area of the specimen (mm^2^).

The experimental cylindrical specimen was 150 mm × 150 mm in size. Before the experiment, the specimen was pretreated, and the upper and lower surfaces were coated with early-strength and high-strength cement paste, and a small amount of fine sand was sprinkled. After pretreatment, the upper and lower surfaces of the specimen were leveled with round steel plates, and the specimen was allowed to stand for 8 h; then the specimen was saturated with water for 24 h. The surface of the saturated specimen was dried, and a circular plate was used to increase the contact area between the pressure plate and the upper surface of the specimen. Afterward, we placed dial indicators at both ends of the loading plate so that the dial indicators at both ends were in contact with the loading plate and were approximately equal to the center of the specimen. We then pre-pressed the specimen twice, and the pre-press load value was half of the maximum load to be applied. After the pre-press, the dial indicator was set to 0 and the original reading was recorded. We then divided the load to be applied into five equal parts, stabilized the pressure for 1 min after each application to the predetermined load, recorded the reading, unloaded it to the initial state, and then recorded the reading. The formula for calculating the resilience modulus (*E*) is *E* = *ph*/*l*, in which *p* is the unit pressure (MPa), *h* is the specimen height (mm), and *l* is the specimen rebound deformation (mm).

#### 2.3.3. Acoustic Detection Test

Ultrasonic testing is a commonly used non-destructive testing method. Based on the influence of internal defects, pores, cracks, and other heterogeneity when ultrasonic waves propagate in the material to be tested, the test would result in different acoustic characteristics, which can be used to evaluate the internal quality and structural status of the cement-stabilized macadam base material. In this study, a non-metallic ultrasonic tester was used to measure the change in the internal ultrasonic propagation velocity of the specimen under different freeze–thaw cycles.

Acoustic emission (AE) technology is a sensitive passive monitoring method. By capturing and analyzing the AE signals generated in the mechanical process of materials, information on internal damage changes can be obtained indirectly. In this study, the acoustic emission signals of the compression failure process of specimens under different freeze–thaw cycles were collected to explore their compression failure damage characteristics.

## 3. Results

### 3.1. Mass Evolution Analysis of Cement-Stabilized Crushed Stone Base with Skeleton Dense Gradation

**Figure 2:**
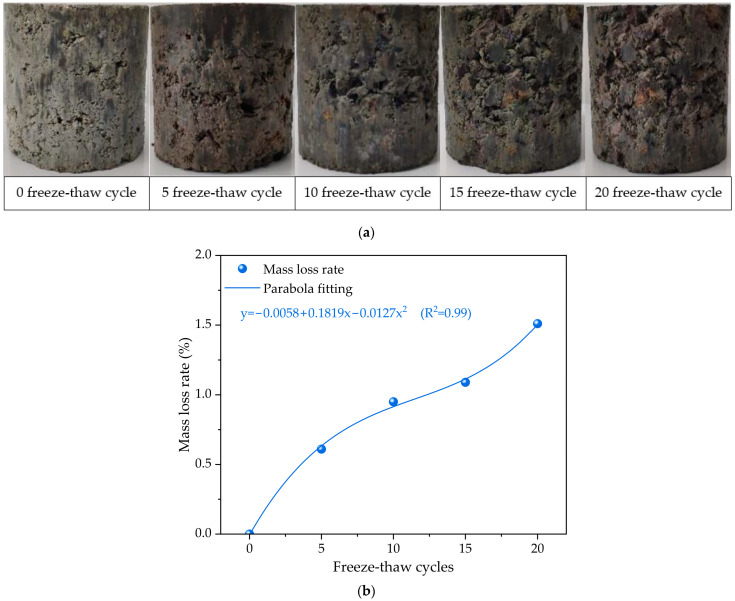
The apparent morphology and mass variation of cement-stabilized crushed stone base with skeleton dense gradation under different freeze–thaw cycles: (**a**) apparent morphology; (**b**) mass variation.

The apparent morphology of the cement-stabilized crushed stone base specimens after undergoing different degrees of freeze–thaw cycles is shown in Figure 2a. Specimens have a relatively smooth surface and intact appearance during the non-freeze–thaw stage, which appears as a porous and non-homogenous external surface due to the skeleton dense gradation. After experiencing five freeze–thaw cycles, the apparent morphology of the cement-stabilized crushed stone base specimens showed almost no significant change, but some dense pores could be observed on the specimen surface. As freeze–thaw damage increases, the surface damage degree of the cement-stabilized crushed stone base specimen gradually intensifies. After experiencing 10–15 freeze–thaw cycles, there is a significant peeling of fine aggregates on the cement-stabilized crushed stone base specimen surface. After 20 freeze–thaw cycles, the cementing materials on the cement-stabilized crushed stone base specimen surface peeled off severely, and the coarse aggregates were exposed to a large area.

Figure 2b illustrates the correlation between the mass loss rate of the cement-stabilized crushed stone base and the number of freeze–thaw cycles. The results indicate that as the number of freeze–thaw cycles increases from 0 to 20, the correlation curve between the specimen mass loss rate and the number of freeze–thaw cycles shows a gradually increasing trend. When the number of freeze–thaw cycles is 5, the mass loss rate reaches approximately 0.61%. As the number of freeze–thaw cycles increases, the mass loss rate increases at a slower rate. When the number of freeze–thaw cycles reaches 15 times, the mass loss rate increases to approximately 1.09%, and the mass loss rate increases to approximately 1.51% at 20 freezing–thawing cycles. Especially, after 15 freeze–thaw cycles, the slope of the mass loss rate curve significantly increased. As the degree of freeze–thaw damage to the cement-stabilized base specimen increases, the mass loss rate curve becomes steeper, indicating a faster rate of change in cement-stabilized crushed stone base mass. The fitting results of the relationship between the mass loss rate of the cement-stabilized base and the freeze–thaw cycling process reveal that they follow a parabola-fitting pattern.

The above results indicate that freeze–thaw cycling has a significant effect on the mass loss rate and apparent morphology of the cement-stabilized base. As the number of freeze–thaw cycles increases, the mass loss rate of the cement-stabilized base gradually increases and the apparent morphology becomes more defective. These structural defects mainly include microcracks and debonding between aggregates and cement paste. This is mainly due to the water content in the cement-stabilized base expanding during freezing as well as contracting during thawing, resulting in microcracks and debonding in the cement-stabilized base structure. These structural defects can significantly reduce the mechanical properties and service life of the cement-stabilized base. Therefore, it is necessary to take measures to reduce the water content in the cement-stabilized base or improve the freeze-resistant ability during construction and use to ensure its service life.

### 3.2. Compressive Strength Evolution of Cement-Stabilized Crushed Stone Base with Skeleton Dense Gradation

**Figure 3:**
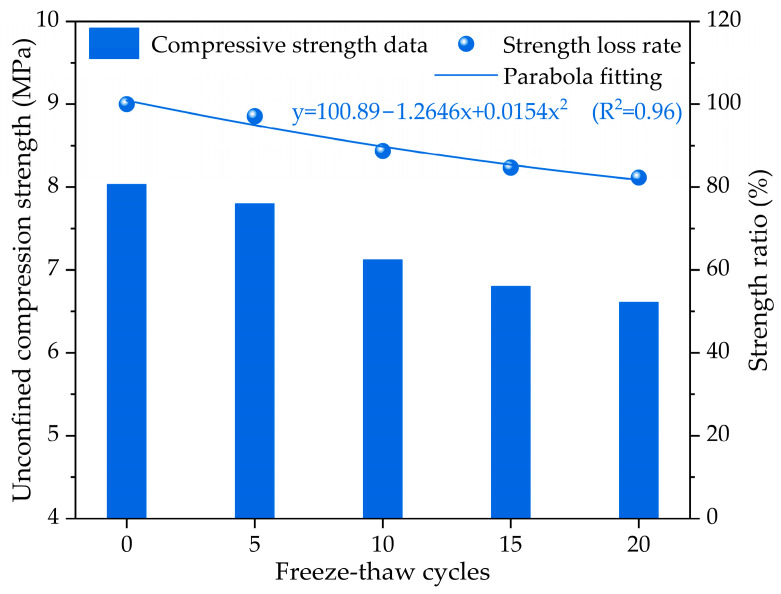
The compressive strength evolution of cement-stabilized crushed stone base with skeleton dense gradation under different freeze–thaw cycles.

The typical relationship of the unconfined compressive strength of the cement-stabilized crushed stone base with skeleton dense gradation between freezing–thawing cycles is plotted in Figure 3. Generally speaking, the compressive strength of the cement-stabilized crushed stone base with skeleton dense gradation decreases with the increase in freeze–thaw cycles. During freeze–thaw cycling, the compressive strength of the cement-stabilized crushed stone base is also affected by various factors such as the water content, temperature, and the presence of freezing-sensitive materials. During 20 freeze–thaw cycles, the strength of the cement-stabilized crushed stone base decreased from 8.03 MPa to approximately 6.61 MPa. However, this trend is not linear. The decrease rate of compressive strength may increase at a faster rate as the number of freeze–thaw cycles increases. The results of the compressive strength ratio show that the compressive strength of the cement-stabilized crushed stone base gradually decreases with the increase in freeze–thaw cycles. When the number of freeze–thaw cycles is 5, the compressive strength ratio reaches approximately 97.1%. As the number of freeze–thaw cycles increases, the compressive strength ratio increases at a faster rate. When the number of freeze–thaw cycles reaches 10, the compressive strength ratio increases to approximately 88.7%. The compressive strength ratios even increase to approximately 84.7% and 82.3% at 15 and 20 freeze–thaw cycles. The fitting results of the relationship between the compressive strength ratio and freeze–thaw cycles reveal that they also follow a parabola-fitting pattern, which is similar to the mass loss rate. As the degree of freezing–thawing damage to the cement-stabilized base specimen increases, the compressive strength ratio curve becomes steeper, indicating a faster rate of change in cement-stabilized crushed stone base strength.

The compressive strength results indicate that freeze–thaw cycling has a significant effect on the compressive strength of the cement-stabilized crushed stone base. As the number of freeze–thaw cycles increases, the compressive strength of the cement-stabilized crushed stone base gradually decreases. The cement-stabilized crushed stone base has less damage in the initial stage and a denser internal pore structure. This is mainly due to the water content in the cement-stabilized crushed stone base expanding during freezing and contracting during thawing, resulting in microcracks and debonding in the cement-stabilized crushed stone base structure. This small amount of damage would lead to structural defects, which can significantly reduce the mechanical properties and service life of the cement-stabilized crushed stone base.

### 3.3. Ultrasonic Wave Velocity Analysis of Cement-Stabilized Crushed Stone Base with Skeleton Dense Gradation

**Figure 4:**
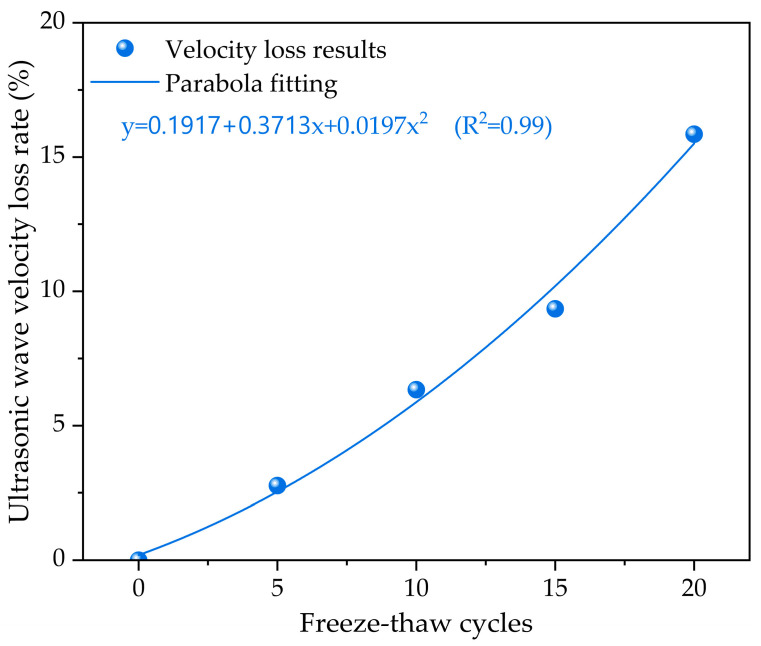
The ultrasonic wave velocity evolution of cement-stabilized crushed stone base with skeleton dense gradation under different freeze–thaw cycles.

Ultrasonic testing is an effective non-destructive method for studying the internal condition of cement-stabilized macadam base materials. Figure 4 illustrates the relationship between the ultrasonic pulse velocity loss rate of concrete and the number of freeze–thaw cycles. It is observed that the ultrasonic wave velocity of the cement-stabilized crushed stone base varies with the number of freeze–thaw cycles. Generally, as the number of freeze–thaw cycles increases, the ultrasonic wave velocity of the base material decreases. This trend can be explained by the propagation principles of ultrasonic waves in the cement-stabilized crushed stone base. Ultrasonic waves propagate in this material through the interaction between the elastic stress field and the density field. When an ultrasonic wave propagates in the base material, it generates a compressive stress field in the material, leading to a density field distribution. The propagation velocity of ultrasonic waves is determined by the elastic modulus and density of the material. During freeze–thaw cycling, repeated freezing and thawing cause microcracks and defects in the structure of the cement-stabilized crushed stone base, reducing the elastic modulus and density of the material and ultimately leading to a decrease in ultrasonic wave velocity.

The ultrasonic wave velocity results reveal that the ultrasonic wave velocity of the cement-stabilized crushed stone base progressively diminishes as the number of freeze–thaw cycles increases. When the number of freeze–thaw cycles reaches five, the ultrasonic wave velocity loss rate reaches approximately 2.77%. As the number of freeze–thaw cycles further increases, the ultrasonic wave velocity loss rate accelerates. When the number of freeze–thaw cycles reaches ten, the ultrasonic wave velocity loss rate climbs to approximately 6.34%. At fifteen and twenty freeze–thaw cycles, the ultrasonic wave velocity loss rates surge to approximately 9.35% and 15.85%, respectively. These findings indicate that freeze–thaw cycling significantly impacts the ultrasonic wave velocity of the cement-stabilized crushed stone base. When conducting polynomial fitting analysis on ultrasonic wave velocity loss rates, a quadratic polynomial equation provides an accurate fit. As the number of freeze–thaw cycles increases, the ultrasonic wave velocity loss rate exhibits a parabolic trend. This trend can be attributed to the gradual increase in microcracks and defects in the cement-stabilized crushed stone base with ongoing freeze–thaw cycling. After a certain number of freeze–thaw cycles, there is a rapid decline in the ultrasonic wave velocity loss rate. In summary, this finding offers valuable insights for monitoring and predicting the mechanical properties and service life of cement-stabilized crushed stone base structures under freeze–thaw cycling conditions.

### 3.4. Resilience Modulus Analysis of Cement-Stabilized Crushed Stone Base with Skeleton Dense Gradation

**Figure 5:**
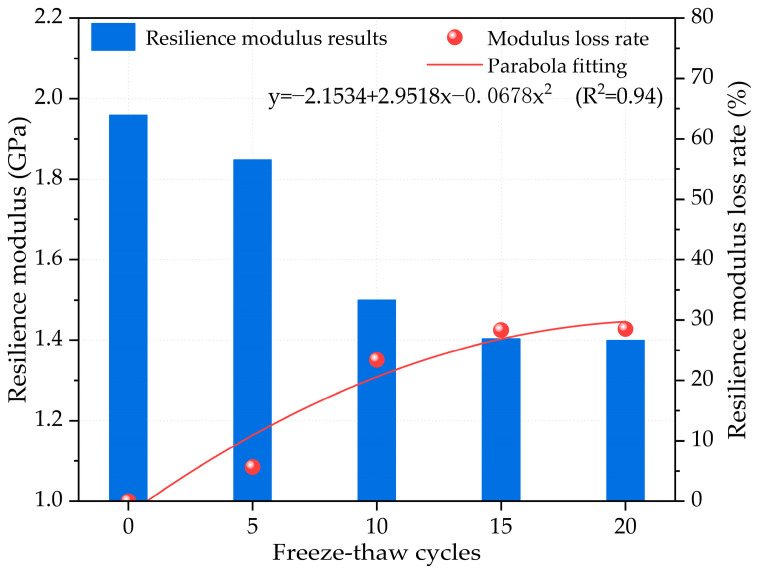
The resilience modulus evolution of cement-stabilized crushed stone base under different freeze–thaw cycles.

The compressive resilience modulus is a key parameter in the design of semi-rigid base materials. Figure 5 shows the typical relationship between the resilience modulus of cement-stabilized crushed stone base exposed to freeze–thaw cycles. Generally, the resilience modulus of a cement-stabilized crushed stone base with skeleton dense gradation decreases as the number of freeze–thaw cycles increases. During freeze–thaw cycling, factors such as water content, temperature, and the presence of freezing-sensitive materials can impact the compressive strength of the cement-stabilized crushed stone base. Water within the cement-stabilized crushed stone base expands when frozen and contracts when thawing. This cyclic expansion and contraction can lead to microcracking and debonding within the material, ultimately reducing its compressive resilience modulus. After more than 20 freeze–thaw cycles, the resilience modulus of the cement-stabilized crushed stone base decreased from 1.96 GPa to approximately 1.40 GPa. However, this trend is not linear, and the decrease rate of the compressive resilience modulus may accelerate as the number of freeze–thaw cycles increases. The results of the compressive resilience modulus loss rate indicate that the resilience modulus of the cement-stabilized base gradually decreases with an increase in freezing–thawing cycles. When the number of freeze–thaw cycles is 5, the resilience modulus loss rate reaches approximately 5.66%. As the number of freeze–thaw cycles increases, the resilience modulus loss rate increases at a faster rate. When the number of freeze–thaw cycles reaches 10, the resilience modulus loss rate increases to approximately 23.41%. The resilience modulus loss rates even increase to approximately 28.34% and 28.54% at 15 and 20 freeze–thaw cycles. The fitted results of the relationship between the resilience modulus loss rate and the freeze–thaw cycles indicate that they also follow a parabola-fitting pattern, which is also similar to the mass loss rate. As the degree of freezing–thawing damage to the cement-stabilized base specimen increases, the resilience modulus loss rate curve becomes steeper, indicating a faster rate of change in the resilience modulus of the cement-stabilized crushed stone base.

The compressive resilience modulus results suggest that freeze–thaw cycling has a significant impact on the resilience modulus of the cement-stabilized crushed stone base. As the number of freeze–thaw cycles increases, the compressive resilience modulus of the cement-stabilized crushed stone base gradually decreases. Initially, the material experiences less damage and has a denser internal pore structure. However, as the number of freeze–thaw cycles increases, damage accumulates, causing a gradual decrease in the compressive resilience modulus. It is important to note that a decrease in the compressive resilience modulus can significantly impact the physical properties as well as the service life of the cement-stabilized crushed stone base. Therefore, it is essential to consider the effect of freeze–thaw cycling on this material when designing infrastructure projects that require its use.

### 3.5. Acoustic Emission Parameter Analysis of Cement-Stabilized Crushed Stone Base under Freeze–Thaw Cycles

**Figure 6:**
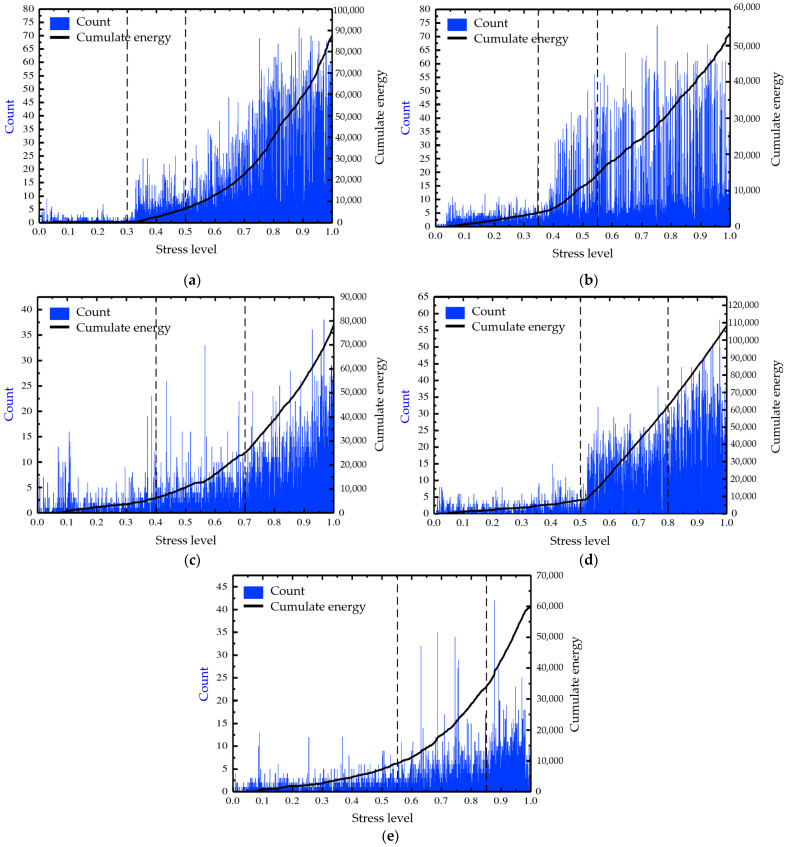
The relationship between AE count and cumulate energy parameters of cement-stabilized crushed stone base with stress level under freeze–thaw cycles: (**a**) 0 freeze–thaw cycles; (**b**) 5 freeze–thaw cycles; (**c**) 10 freeze–thaw cycles; (**d**) 15 freeze–thaw cycles; (**e**) 20 freeze–thaw cycles.

Figure 6 illustrates the correlation between the AE parameters and stress levels under different freezing–thawing cycles. As plotted in Figure 6, the entire failure and cracking process of the cement-stabilized base with skeleton dense gradation under compressive load can be divided into three stages.

Stage I—Damage initial stage: the low-level stress only changes slightly, but no new cracks are produced, and the damage is in the stage of slight loss. The AE cumulate energy–stress level curve of the cement-stabilized crushed stone base shows a linear behavior. Stage II—Damage stationary stage: the internal cracks of the cement-stabilized macadam specimens are increasing, and a small number of micro-cracks appear on the surface. The accumulated energy begins to increase at this stage, and the increasing trend gradually becomes faster. The AE count begins to increase, but the overall AE count is relatively stable. The AE cumulate energy–stress level curve of the cement-stabilized crushed stone base shows an obvious nonlinear behavior. Stage III—Damage failure stage: the performance of the specimen itself deteriorated seriously at the later stage of damage, showing a brittle failure mode of sudden failure. The AE count increased rapidly, and the AE cumulate energy–stress level curve of the cement-stabilized crushed stone base showed a sharp increase.

From the relationship between the AE parameters and stress level under different freeze–thaw cycles, it can be seen that the period of stage I would increase with the increase in freeze–thaw cycles. This is because the freeze–thaw process causes damage to the internal microstructure of the cement-stabilized base, and there are more and more internal defects. It needs a relatively larger stress level to show macro cracks for the cement-stabilized base with freezing–thawing cycles. What is interesting about the relationship in Figure 6 is that the period of stage III is shortened with the increase in freezing–thawing cycles, which could be attributed to the internal defects and cracks expanding rapidly under the freeze–thaw action. These changes can be used to evaluate the damage status and performance degradation of the cement-stabilized crushed stone base during freeze–thaw action.

## 4. Discussions

The freeze–thaw damage characteristics of the cement-stabilized crushed stone base with skeleton dense gradation, as explored in this study, reveal a complex interaction between semi-rigid base material properties and freeze–thaw environmental conditions. The above observed trends in mass loss, compressive strength, resilience modulus, ultrasonic wave velocity, and AE parameters provide valuable insights into the behavior of the cement-stabilized crushed stone base with skeleton dense gradation under freeze–thaw cycling. Firstly, the gradually increasing trend in the mass loss rate with increasing freeze–thaw cycles indicates that the material is undergoing degradation over time. The steepening curve after 15 freeze–thaw cycles suggests that the rate of degradation accelerates as the material undergoes more cycles. This is consistent with the observed decrease in compressive strength and resilience modulus, which both follow a parabolic trend [34]. These findings suggest that the material’s resistance to freeze–thaw damage decreases as the number of freeze–thaw cycles increases [35]. The decrease in compressive strength is particularly significant as it directly affects the structural integrity of the road base. The accelerating decrease in strength with increasing freeze–thaw cycles could lead to premature failure of the road, necessitating costly repairs and replacements [36]. The resilience modulus, a metric that quantifies a material’s capacity to recover from deformation, serves as an indicator of elastic properties’ degradation. Specifically, a decrease in the resilience modulus signifies a loss of these elastic characteristics [37,38]. Repeated cycles of water freezing and thawing result in the erosion of small particles, thereby causing a gradual reduction in material mass [39].

The observed parabolic trends in mass loss, compressive strength, and resilience modulus are indicative of a nonlinear relationship between freeze–thaw cycling and material degradation. This is further supported by the decrease in ultrasonic wave velocity, which is a measure of material stiffness, reflecting its microscopic pore structure [40]. The parallel trends in mechanical performances and ultrasonic wave velocity suggest that the material’s stiffness and elasticity are compromised by freeze–thaw cycling. Freeze–thaw cycles cause water absorption and desorption within the pores and voids of the cement-stabilized crushed stone. As water freezes, it expands, exerting pressure on the surrounding material [41]. This pressure, along with the formation of ice crystals, can lead to the disintegration of weak bonds and the creation of new cracks or the enlargement of existing ones [42]. Over time, this cumulative damage results in a significant loss of material integrity. As cracks form and expand, the material’s ability to withstand compressive loads weakens. This nonlinearity variation trend is important to consider in the design and maintenance of roads constructed with a cement-stabilized crushed stone base [43,44]. The findings of this study highlight the need for a better understanding of the freeze–thaw damage characteristics of cement-stabilized crushed stone bases. Future research should focus on developing improved materials and design strategies that can mitigate the effects of freeze–thaw cycling, thereby extending the lifespan and durability of roads.

## 5. Conclusions

Based on the mechanical performances and acoustic detection tests, the study has provided valuable insights into the mechanisms and processes of freeze–thaw damage in cement-stabilized crushed stone bases with skeleton dense gradation. The study’s findings are summarized as follows:

(1) The mass loss rate of the cement-stabilized crushed stone base shows a gradually increasing trend with freezing–thawing cycles increasing, in which the curve steepens significantly after 15 freeze–thaw cycles, following a parabola-fitting pattern relationship.
(2) The compressive strength of the cement-stabilized crushed stone base with skeleton dense gradation decreases with freezing–thawing cycle increasing, following a parabola-fitting pattern. The decrease rate may accelerate as the cycles increase.
(3) The ultrasonic wave velocity of the cement-stabilized crushed stone base decreases with increasing freeze–thaw cycles, exhibiting a parabolic trend. This decline can be attributed to microcracks and defects, offering insights for monitoring and predicting the structure’s service life.
(4) The resilience modulus decreases with increasing freeze–thaw cycles for the cement-stabilized crushed stone base, following a parabolic trend. This reduction can be attributed to microcracking and debonding within the material.
(5) The damage to the cement-stabilized crushed stone base progresses through three stages: initial, stationary, and failure according to AE parameters. The stage I duration increases with freeze–thaw cycles, while the stage III duration decreases, reflecting internal defects and crack growth under freeze–thaw cycling.

Prior research may have been limited to general materials or specific freeze–thaw damage facets. However, this study innovates by offering a comprehensive examination of the freeze–thaw damage process, accounting for the distinct features of cement-stabilized crushed stone with dense skeleton gradation. The insights gained not only deepen the comprehension of damage mechanisms but also facilitate precise lifespan predictions for road structures in future work, enabling cost-effective maintenance and sustainable infrastructure development. We also recognize the need for further investigation to fully understand the long-term effects of freeze–thaw cycling and go deeper into microscopic mechanism analysis.

## Figures and Tables

**Table 1:** The performance indexes of cement.

Performance Indexes	Results	Standard (GB 175-2007) [30]
Initial setting time (h)	2	≥0.75
Final setting time (h)	3	≤10
3 d compressive strength (MPa)	25	≥21
28 d compressive strength (MPa)	53	≥42.5
3 d flexural strength (MPa)	5.2	≥4.0
28 d flexural strength (MPa)	8.5	≥6.5

**Table 2:** The performance indexes of coarse aggregates.

Performance Indexes	Results	Standard (GB 175-2007) [30]
Apparent relative density	2.766	/
Water absorption (%)	1.24	/
Needle-like content (%)	10.6	≤18
Crushing value (%)	21.5	≤22

**Table 3:** The performance indexes of fine aggregates.

Performance Indexes	Results	Standard (GB 175-2007) [30]
Apparent relative density	2.682	/
Fineness modulus	2.85	/
Water absorption (%)	1.72	/
Plasticity index	10.6	≤17
Liquid limit (%)	23.2	/
Plastic limit (%)	12.6	/

**Table 4:** The percentage of passing through screening of each grade aggregate.

Sieve Size (mm)	20~30 mm (%)	10~20 mm (%)	5~10 mm (%)	0~5 mm (%)
31.5	100	100	100	100
26.5	82.19	100	100	100
19	33.63	75.67	100	100
16	1.27	58.01	100	100
13.2	0.38	36.45	100	100
9.5	0	9.73	100	100
4.75	0	0.44	50.93	92.28
2.36	0	0	23.36	74.66
1.18	0	0	15.31	46.15
0.6	0	0	10.85	29.12
0.3	0	0	6.11	19.01
0.15	0	0	4.15	7.72
0.075	0	0	2.65	2.97

## Data Availability

Data are contained within the article.
